# The sensory and flavor characteristics of Shaoxing Huangjiu (Chinese rice wine) were significantly influenced by micro‐oxygen and electric field

**DOI:** 10.1002/fsn3.2531

**Published:** 2021-09-10

**Authors:** Chi Shen, Hongyi Zhu, Wenxia Zhu, Yimeng Zhu, Qi Peng, Nabil I. Elsheery, Jianwei Fu, Guangfa Xie, Huajun Zheng, Jiongping Han, Baowei Hu, Jianqiu Sun, Peng Wu, Yuyan Fan, Dula Bealu Girma

**Affiliations:** ^1^ National Engineering Research Center for Chinese CRW (branch center) Shaoxing University Shaoxing China; ^2^ California Institute of Food and Agricultural Research University of California Davis CA USA; ^3^ Agricultural Botany Department Faculty of Agriculture Tanta University Tanta Egypt; ^4^ College of Biology and Environmental Engineering College of Shaoxing CRW Zhejiang Shuren University Hangzhou China; ^5^ School of Medicine Shaoxing University Shaoxing China; ^6^ School of Environmental Science and Engineering Suzhou University of Science and Technology China

**Keywords:** Chinese rice wine, electric field, flavor characteristic, free amino acids, micro‐oxygen, sensory profile

## Abstract

In order to improve the high cost of equipment and difficult management caused by the natural aging of Chinese rice wine (Huangjiu), micro‐oxygen (MO) and electric field (PEF) technology are used to accelerate the aging of Huangjiu. The results showed that micro‐oxygen and electric field have a significant effect on the sensory characteristics and flavor characteristics of Huangjiu. Compared with the naturally aged Huangjiu, the flavor compounds of Huangjiu treated with micro‐oxygen and electric field increase significantly. Based on principal component analysis, Huangjiu processed at 0.35 mg L/day or 0.5 mg L/day combined electric field exhibited similar flavor to the natural aged Huangjiu, which was highly associated with long‐chain fatty acid ethyl esters (C13–C18). Moreover, partial least squares regression demonstrated that sensory attributes of cereal aroma and astringency were highlighted after aging time, while fruit aroma, continuation, and full body were dominant after micro‐oxygen and electric field treatment. Micro‐oxygen and electric field effectively enhanced the quality of Huangjiu, which could be applied in other alcoholic beverages.

## Graphical abstract

1. Artificial aging technology affects the sensory and flavor changes of Huangjiu.2. Micro‐oxygen and electric field mixed treatment improve the quality of Huangjiu.3. Artificial aging technology effectively improves the sensory properties of Huangjiu.

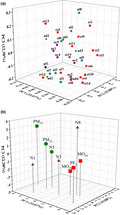

## 1 INTRODUCTION

Chinese rice wine (Huangjiu), a traditional Chinese alcohol beverage, is popular among Chinese customers. Huangjiu, as a traditional alcoholic beverage with high nutritional value and unique flavor, has a history of several centuries in East Asia (Shen et al., 2012). Huangjiu is made from wheat and other grains as raw materials, using Wheat Qu, rice Qu, or alcoholic medicine as saccharifying agent, and after steps such as steamed rice, yeast fermentation, fried wine, and storage (Ren et al., 2016; Shen et al., 2018). The taste of young Huangjiu is rough, and the aroma is insufficient. However, natural aging needs a long storage time, large space, and high equipment costs, which is a major problem to limit the Huangjiu market (Jiao, Xu, & Jin, 2017). For this reason, people are exploring ways to speed up the aging of Huangjiu.

Micro‐oxygen technology refers to a technology that meets the oxygen demand of various chemical and physical reactions in the wine body through different oxygen levels (Ming et al., 2018; Yang et al., 2019). In recent years, it has been involved in sludge digestion, water quality improvement, and biological denitrification (Duc & Kumar, 2018; Zhang et al., 2019). However, little is known about the efficacy of micro‐oxygen technology in the treatment of Huangjiu. Electric field treatment is an emerging nonthermal sterilization technology in the fields of food sterilization, preservation, and ingredient extraction (Caminiti et al., 2011; Yang et al., 2019b). Its processing cycle is short, heat production is small, and it can effectively protect the nutrients of food (Dalvi‐Isfahan et al., 2016; Gabri et al., 2017). Besides, electric field treatment on wine flavor compounds has produced conclusive evidence (Teusdea et al., 2017), indicating that it can improve the sensory properties of fermented red wine. However, the effect of electric field treatment on the quality of Huangjiu has rarely been studied, although Huangjiu has a broad sales prospect in the wine market. During Huangjiu brewing, the degradation of raw materials by cofermentation with lactic acid bacteria, fungi, and yeast results of the production with a large number of amino acids, proteins, oligosaccharides, vitamins, and mineral elements (Lv et al., 2016). Amino acids are nutrient components of Huangjiu and the precursors for aroentma compounds, which contribute to the formation of the flavor of Huangjiu (Miao et al., 2015; Shen et al., 2010). Several studies have reported that applying appropriate pretreatment, fermentation, or saccharification starters could greatly contribute to the aroma and flavor profiles of wines (Takahashi et al., 2017; Wu et al., 2015). However, the flavor compounds in Huangjiu need longer aging time to accumulate, so it is suggested to use micro‐oxygen and electric field treatment instead of traditional natural aging to produce high quality Huangjiu. Researchers and winemakers need to understand the effect of aging on the flavor properties of Huangjiu in different accelerated aging treatments.

As amino acids and aroma were important in Huangjiu, this study aimed at analyses the differences of brewing characteristics, free amino acids, flavor, and sensory characteristics of Huangjiu under three treatment conditions of natural aging, micro‐oxygen, and electric field. This study could provide advice for winemakers to choose appropriate accelerated aging treatments to produce high quality Huangjiu or other alcoholic beverages.

## 2 MATERIALS AND METHODS

### 2.1 Huangjiu sample material

Huangjiu with different aging time: Provided by Zhejiang Guyue Longshan Shaoxing Wine Co., LTD. The Huangjiu was fermented in the cellar at constant temperature and aged in pottery jars. The same original wine age is 1, 3, 5, and 8 years Huangjiu, and the wine sample is put in a clean plastic bottle, stored in a 4°C constant temperature refrigerator, ready to use.

#### 2.1.1 Micro‐oxygen treatment

The oxygen content into Huangjiu is controlled by the flowmeter and then used the syringe to inject oxygen into Huangjiu, with a timer to control the interval cycle of predetermined oxygen content. The micro‐oxygen has combined the advantages of both aerobic (Mass concentration of dissolved oxygen >1.0 mg/L) and anaerobic (Mass concentration of dissolved oxygen <0.3 mg/L and does not contain nitrate oxygen), usually refers to the environmental dissolved oxygen mass concentration in the reactor is 0.3 ~ 1.0 mg/L (Duc & Kumar, 2018), while the daily dissolved oxygen concentration of Huangjiu in the pottery jars is 0.35 mg/L. The method of adding oxygen from bottom to top is adopted to make oxygen fully contact with liquid and achieve better oxidation effect. In this experiment, the amounts of oxygens added and the action time were used as variables to observe the effects on various compounds in Huangjiu. Accelerated aging treatments for 60 days were performed on micro‐oxygen 0.35 mg L/day (MO36) and for 60 days on micro‐oxygen 0.5 mg L/day (MO56). The temperature of micro‐oxygen treatment should be kept at 15 ~ 18°C, because the temperature will affect the solubility of oxygen in Huangjiu. Second, there is a certain height requirement for the oxygenated container, so as to ensure that the tiny bubbles of oxygen can be fully dissolved in Huangjiu, so the container should be at least 2 m high.

#### 2.1.2 The electric field treatment

The electric field treatment of Huangjiu uses a static pulse treatment chamber. The pulse discharge waveform adopts exponential attenuation wave, and the electrode is stainless steel parallel electrode. Considering the effect of electric field strength on the features of Huangjiu, the wine samples were processed under the optimal parameters of electric field strength of 2 kv/cm, pulse number of 36, and frequency of 0.5 Hz. The samples of traditional natural aged for 1 year (N1), natural aged for 3 years (N3), natural aged for 5 years (N5), natural aged for 8 years (N8), 2 kv/cm (PF2), 0.35 mg L/day 2 kv/cm (PM32), and 0.5 mg L/day 2 kv/cm (PM52) were tested after different treatments.

### 2.2 Enological properties of Huangjiu

The total sugar content was determined by dinitrosalicylic acid method. Total acidity (in terms of lactic acid) and alcohol content were measured by Chinese standard method (GB/T 13662‐2018). The analysis carried out by high‐performance liquid chromatography (Waters e2695‐Empower System Waters Corporation, Milford, MA) equipped with exclusion column (Carbomix H‐NP, 300 mm × 7.8 mm, Sepax Technologies, Inc., Newark, DE) at 55°C with a mobile phase of sulfuric acid (2.5 mM) at a flow rate of 0.6 ml/min. In order to determine the total nitrogen, 5 ml of wine sample and catalyst was digested at 420°C for 150 min and analyzed by an automatic Kjeldahl apparatus (Kjeltec 8400; FOSS., Hilleroed, Denmark). Turbidity was measured by nephelometric analysis using a WGZ‐1 turbidity meter (WGZ‐1; Shanghai Xinrui Instrument Co., Ltd, Shanghai, China) and calibrated with a standard solution. The turbidity index of Huangjiu samples is expressed in units of turbidity (NTU).

The red, orange, and yellow produced by the saccharifying agent have the maximum absorption at 510, 465, and 410 nm, respectively. Appropriate dilutions of wine samples were measured for absorbance at 510, 465, and 410 nm with a spectrophotometer (V‐1800; Shanghai Meipuda Instrument Co., Ltd, Shanghai, China). The color value was defined as absorbance unit at maximum absorption wavelength multiplied by a dilution factor per milliliter of wine sample (U/ml).

### 2.3 Analysis of free amino acids

According to the method described in Xia et al (Xia et al., 2016), the previously filtered Huangjiu sample was precipitated at 4°C for 2 hr with an equivalent amount of 10% sulfosalicylic acid to remove the large peptides and then centrifuged at 12,000 g for 20 min. The 20 μl supernatant was injected into t/L8900 automatic amino acid analyzer (Hitachi, Tokyo, Japan). Determination of free amino acids in the Huangjiu sample was calculated by calibrating with standard amino acids.

### 2.4 Volatile flavor compounds

The 5 ml Huangjiu sample was diluted and placed in a 20 ml headspace glass vials. Then, add 2 g sodium chloride and 10 μl internal standard of 4‐methyl‐2‐pentanol (250 μg/ml in absolute ethanol) (Yang et al., 2017). The fiber (50 μm DVB/CAR/PDMS) in a solid‐phase microextraction (SPME) device (Supelco, Bellefonte, PA) was inserted into the vials and then extracted at 50°C for 30 min. The volatile compounds were then desorbed at 250°C into the GC inlet with an auto‐sampler for 7 min. A GC‐MS system (SCION SQ‐456; Bruker Daltonics Inc., Billerica, MA) equipped with a DB‐WAX column (60 m × 0.25 mm × 0.25 μm; Agilent Technologies, Santa Clara, CA) and programmed from 40°C (holding for 3 min) to 210°C at 6 °C/min and then 210°C to 230°C at 8 °C/min (holding for 15 min). Electron ionization energy: 70EV; Ion source temperature: 220℃; Helium carrier gas: 1 ml/min (Jung et al., 2014).

The Kovats indices (KI) of unknown compounds were determined by sample injection with a homologous series of alkanes (C7–C30). The extracted volatile compounds were compared with the mass spectral libraries (NIST 1.6 and Wiley 6.0), and the mass spectra and KIs of the tentatively identified compounds were compared with those of authentic standards for positive identification. The content of volatile compounds was determined by internal standard method. After preliminary identification of mass spectra and KIs in the literature, semi‐quantitative data of volatile compounds are calculated according to the following formula (Mo et al., 2012):

$$C\left(\mu g/\mathrm{ml}\right)=\;\frac{A_{C}}{A_{\mathrm{is}}}C_{\mathrm{is}}\left(\mu g/\mathrm{ml}\right).$$

C is the relative concentration of the analyte; C_is_ is the final concentration of the internal standard; A_c_ is the peak area of the analyte; A_is_ is the internal standard of the peak area.

### 2.5 Sensory evaluation

Sensory evaluation of Huangjiu was conducted by a training panel of 10 judges (five males and five females 20–50 years old). The judges were selected and trained according to ISO 8586‐1 (ISO, 1993). A total of 12 descriptors (appearance: yellowness, redness, and turbidity; aroma: alcohol, fruit, and cereal; taste: sweet, sour, and bitter; mouthfeel: astringency, continuation, and full body) were generated, to characterize the sensory properties of Huangjiu (GB/T 13662‐2018). During the training, guide the judges to learn the Huangjiu scoring standards (Table 1). The reference table (Table 1) used to evaluate the sensory level of Huangjiu was included in the training and wine‐tasting tests.

**TABLE 1:** Huangjiu scoring standard

Projects	Standards
Color (10 scores)	Orange yellow, brown, orange red, or Huangjiu should have the color, transparent clear luster (10 scores)
	Transparent and clear, gloss slightly poor (8 ~ 9 scores)
	Slightly turbid, transparent, but poor luster (4 ~ 7 scores)
	Turbidness, lack of luster, lack of Huangjiu color (0 ~ 3 scores)
Perfumed (25 scores)	Has the Huangjiu special aroma, the aroma is mellow (25 scores)
	Has an aroma of Huangjiu, full bodied but not intense (22 ~ 24 scores)
	Has the aroma of Huangjiu, but the mellow aroma is not obvious (15 ~ 21 scores)
	Lack of Huangjiu aroma, slight other aroma (8 ~ 14 scores)
	Have complex or other unpleasant smells that Huangjiu should not have (0 ~ 7 scores)
Tastes (50 scores)	Fresh or sweet, mellow, soft, and refreshing (50 scores)
	Delicious (sweet), mellow, and refreshing, but not soft enough (45 ~ 49 scores)
	Delicious (sweet), slightly refreshing, light wine taste (35 ~ 44 scores)
	The wine is light, slightly bitter, and ripe (25 ~ 34 scores)
	The wine is not mellow and has a bitter, spicy taste (15 ~ 24 scores)
	Tasteless, bitter, sour (10 ~ 14 scores)
	Rancidity and mixed taste (0 ~ 9 scores)
Style (15 scores)	With the unique style of Huangjiu, wine flavor coordination (15 scores) has the Huangjiu's unique style, and the wine's flavor is generally coordinated (12 ~ 14 scores)
	The style is not obvious, and the flavors of the wine are generally harmonious (5 ~ 11 scores)
	The flavors of the wine are discordant and spicy (0 ~ 4 scores)

There was a uniform source of lightening, absence of noise, and distracting stimuli in the training and wine‐tasting test. Put different Huangjiu samples (15 ml) into transparent glass, cover with petri dishes, and label them randomly. Provide drinking water to panelists to reduce taste disturbance.

### 2.6 Statistical analysis

Perform triplicate chemical analysis for each Huangjiu sample, and the result was expressed as mean value ± standard deviation (*SD*). One‐way ANOVA, followed by Duncan's test, was performed to analyze the significant differences between data. The mean difference was considered significant at *p* <.05. PCA using a correlation matrix with no rotation was performed to research the relationship between different processing method wine samples and volatile compounds. PLSR studied to the relationship among wine samples, volatile flavor compounds, and sensory descriptors. The difference in amino acids was visualized as a heat map using Cluster Ver.3.0 with TreeView (both University of California). Linear normalization, average linkage, and hierarchical clustering were applied for the cluster analysis. SPSS 26.0 (SPSS Inc., Chicago, IL), Origin 9.5 (Origin Lab Inc., Northampton, MA), Excel 2020 (Excel Communications, MA), and Microsoft Office 2020 (Microsoft Corp., Redmond, WA) were used to deal with the GC‐MS and chemical analysis data.

## 3 RESULTS AND DISCUSSIONS

### 3.1 Effects on enological properties of Huangjiu

Characteristics of naturally aged, micro‐oxygen and electric field treated Huangjiu (total sugar, total acidity, amino acid nitrogen, and ethanol) in Table 2. After natural aging, the acidity of Huangjiu increased and the alcohol content decreased. Due to the inevitable loss of volatile alcohols and aldehydes caused by aging, the total acid content of naturally aged Huangjiu was significantly lower than that of the slightly oxygenated Huangjiu. Ethanol is closely associated with the oxidation and esterification of Huangjiu (Wang et al., 2014). Compared with the natural aged Huangjiu, the decline trend of ethanol in Huangjiu was relatively flat after electric field treatment, indicating that electric field treatment promoted the production of ethanol. Among the aging‐treated Huangjiu, N8 has the lowest ethanol content, indicating that part of the ethanol will volatilize during the aging process. The esterification of Huangjiu in the aging process is also one of the reasons for the decrease of ethanol content (Xu et al., 2015). The proportion of alcohol, acid, and ester can be changed by controlling the electric field intensity, so as to accelerate the aging process. After the electric field treatment, the change trend of amino acid nitrogen was relatively stable, and the total sugar content decreased, while the total acid was just the opposite. MO56 exhibited the highest absorbance at 420 nm (0.685), followed by MO36 (A420 = 0.617), indicating that micro‐oxygen treatment could markedly improve the speed of Maillard reaction in Huangjiu. Since electric field treatment can improve the absorbance of Huangjiu (Rajha et al., 2016), the sugars and amino acids in Huangjiu accelerate Maillard reaction under the action of electric field, which promotes the reduction of total sugar content. The aldehydes in Huangjiu are oxidized to acids which increase the total acidity (Tian et al., 2016). Nevertheless, the electric field treatment maintained the total acid content of Huangjiu.

**TABLE 2:** Different treatment of Huangjiu brewing characteristics

	Total sugar (g/L)	Total acidity (g/L)	Amino acid nitrogen (g/L)	Ethanol (%Vol)
N1	23.61 ± 0.0700^a^	4.58 ± 0.05568^a^	0.8 ± 0.04359^b^	16.4 ± 0.05000^a^
N3	22.61 ± 0.19698^d^	4.75 ± 0.06083^bc^	0.8 ± 0.03606^a^	15.8 ± 0.01000^c^
N5	21.63 ± 0.04583^e^	4.81 ± 0.04583^cde^	0.83 ± 0.07000^ab^	15.4 ± 0.07211^f^
N8	21.14 ± 0.03606g	4.89 ± 0.11533^e^	0.86 ± 0.04000^a^	15.2 ± 0.05568^h^
MO_36_	23.21 ± 0.03000_b_	4.57 ± 0.05292^a^	0.79 ± 0.01000^b^	16.3 ± 0.01000^b^
MO_56_	23.02 ± 0.08185_c_	4.69 ± 0.01732^b^	0.81 ± 0.02646^b^	15.6 ± 0.06245^e^
PF_2_	22.58 ± 0.05292_d_	4.77 ± 0.02646^bcd^	0.81 ± 0.03464^b^	15.7 ± 0.05568^d^
PM_32_	21.58 ± 0.01732^ef^	4.85 ± 0.02646^cde^	0.84 ± 0.05000^ab^	15.3 ± 0.02646^g^
PM_52_	21.42 ± 0.06083^f^	4.86 ± 0.01000^de^	0.85 ± 0.04000^ab^	15.2 ± 0.01000^h^

Note

Values are significantly different (*p* <.05) labeled by different superscript letters within a column.

Significant differences of total sugar, total acidity, and ethanol were observed among different accelerated aging treatments wine, indicating that micro‐oxygen and electric field treatment had an important impact on the flavor components of Huangjiu. Appropriate amount of oxygen can improve the taste of rice wine, but too high oxygen content is not conducive to the improvement of the quality of rice wine. On the one hand, acetaldehyde participates in acetaldehyde bridge reaction and forms acetaldehyde polyphenol polymer with polyphenols. On the other hand, in the process of rice wine aging, through oxidation, reduction, esterification, and other chemical reactions, the aging aroma was formed, which became more and more strong with the passage of time. It is worth noting that if the concentration of dissolved oxygen is too high, the strong oxidation conditions will promote the oxidative polymerization of phenols and produce free radicals, which will lead to the oxidation taste of liquor and reduce the quality of liquor. The technical principle of aging rice wine by high voltage pulsed electric field is to use the energy provided by high voltage pulsed electric field to change the wine body into a strong oxidation state after anodization and accelerate a series of reaction processes such as oxidation–reduction and esterification. PM52 has the lowest ethanol content among the five aging‐treated Huangjiu (Table 2), suggesting that oxygenation was 0.5 mg L/day at 2 kv/cm might promote oxidation and esterification reactions in Huangjiu.

### 3.2 Effect of total free Amino acids on Huangjiu

**FIGURE 1:**
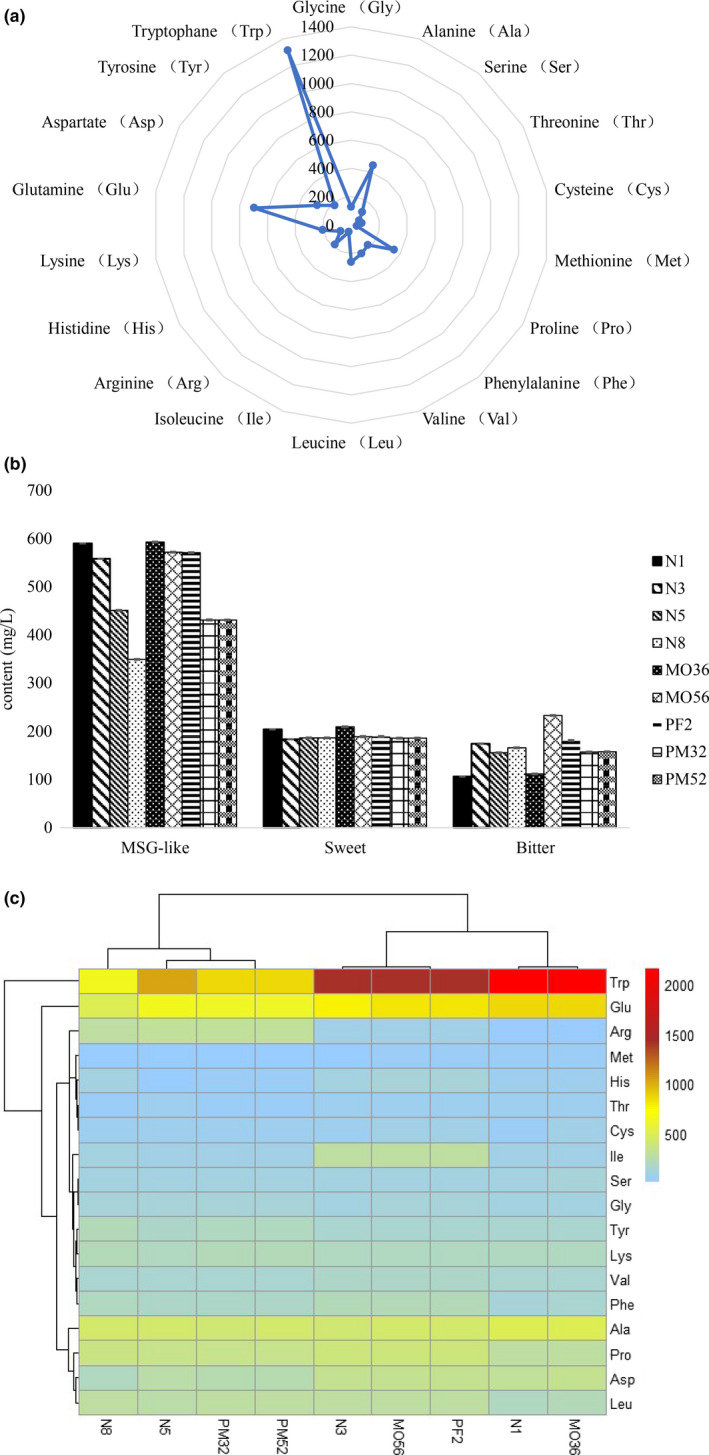
Effects of different accelerated aging treatments on total free amino acid content. (a) Content of free amino acids in Huangjiu (mg/L). (b) Total content of free amino acids with taste characteristic in different treatments wines. (c) Difference in amino acid content between natural aging and accelerated aging treatment of Huangjiu

The effect of different accelerated aging treatments on the total free amino acid content is shown in Figure 1. Eighteen common free amino acids were quantitatively analyzed in wine samples.

Based on the study of Komata (1969), the taste properties of free amino acids could be divided into several categories, which were MSG‐like (Asp and Glu), sweet (Ala, Gly, Ser, and Thr), and bitter (Arg, His, Ile, Leu, Met, Phe, and Val). In a short time, the sweet and MSG‐like components of Huangjiu treated with micro‐oxygen and electric field could reach the aging level in a short time, with different contents, but the variation trend was the same. The total content of sweet and MSG‐like components in MO36 was 11.2% and 27.3% higher than that in PM52, indicating that 0.35 mg L/day (60 days) of micro‐oxygen was more favorable to sweet and fresh amino acids (Figure 1b). In terms of bitter amino acids, the contents of Huangjiu treated by accelerating aging showed a general trend of decline. Compared with the natural aged Huangjiu, micro‐oxygen and electric field treatment reduce the bitterness of the wine and harmonize the natural flavor of Huangjiu. The difference of 18 amino acids between naturally aged Huangjiu and other Huangjiu treated with aging is shown in Figure 1c. The Browning reaction resulted in the decrease of amino acid content in natural aged Huangjiu, which was accelerated by electric field treatment (Ming, Qiu, et al., 2018). Although the content of free amino acids in Huangjiu had a similar changing trend under different micro‐oxygen and electric field treatment conditions, the content of 18 individual free amino acids in four kinds of Huangjiu with micro‐oxygen was different. One amino acid in N1 was found with an increase above 1000 mg/L, one in N3, one in N5, zero in N8, one in PF2, one in MO36, one in MO56, zero in PM32, and zero in PM52 (Figure 1c). The contents of Pro, Gly, Phe, Tyr, and Cys in PM52 were significantly increased, indicating that the aging effect of Huangjiu treated at 0.5 mg L/day 2 kv/cm was better. Some accelerated aging treatments reduced the content of Thr, Glu, Ala, and Met, while the sweetness was increased by the combination of micro‐oxygen and electric field. While other treatments reduced the content of bitter amino acids such as Val, Ile, His, and Leu (Figure 1c), Met, Ile, Gly, Asp, Phe, Tyr, Arg and so on are more sensitive to accelerated aging treatments. Since free amino acids are precursors of flavor compounds (Krivoruchko & Nielsen, 2015), we speculated that these 8 free amino acids were highly correlated to the complex synthesis of flavor compounds in Huangjiu and great significance to the overall aroma of Huangjiu.

### 3.3 Effects on volatile flavor compounds of Huangjiu

The volatile flavor compounds strongly influenced the typical taste and odor of Huangjiu (Chen et al., 2013; Yu et al., 2012). Although volatile flavor compounds were mainly synthesized by microorganisms and enzymes, traditional natural aging greatly affects the flavor profiles in the final Huangjiu (Ren et al., 2019). The determination of volatile flavor substances in natural aged Huangjiu and other five kinds of Huangjiu treated with aging treatment showed that the content of volatile flavor components in N8 (1,135,951.17 mg/L) was higher than that in N1 (979,601.23 mg/L) (Table 3). A total of 33 compounds were studied and analyzed in the final wine, including 14 esters, 8 alcohols 4 aldehydes, 5 fatty acids, and γ‐nonanoic lactone and phenol. Almost all of those compounds have been detected in Huangjiu, and the concentration distributions of flavor compounds were consistent with previous research results.(Jung et al., 2014; Tian et al., 2016). It can be seen from Table 3 that the total content of volatile compounds in the Huangjiu treated with micro‐oxygen (0.5 mg L/day) and electric field was 1,122,972.96 mg/L, which was higher than that in the naturally aged Huangjiu. In addition to the instability of the 1‐year‐old Huangjiu system, the longer the natural aging time was, the more volatile flavor substances were found in Huangjiu, followed by N8 (1,135,951.17 mg/L) and N5 (1,135,712.79 mg/L). In terms of micro‐oxygen and electric field treatment, when the electric field intensity of Huangjiu with oxygen content of 0.5 mg L/day is 2 kv/cm, the content of flavoring substances in Huangjiu could reach the level of natural aging for 5 years. The overall flavor yield of PM32 (1,152,000.82 mg/L) Huangjiu was the highest, and its flavor content increased by 14.97% compared with the natural 1‐year‐old Huangjiu. Compared with the Huangjiu treated with electric field only, the content of flavor substances in PM32 and PM52 increased by 21.81% and 19.79%, indicating that the combination of micro‐oxygen and electric field had greater influence on the flavor of Huangjiu. Single micro‐oxygen or electric field treatment can improve the quality of rice wine and accelerate the aging of rice wine, but the experimental results show that only some indexes have positive changes. The combination of micro‐oxygen and electric field treatment can select the optimal micro‐oxygen through manual intervention and combine with electric field to control the speed of oxidation–reduction, esterification, and other reactions, so it is better than single aging treatment.

**TABLE 3:** Changes of volatile compounds in Huangjiu after different treatments (mg/L)

Types	English names	Serial number	Volatile component content (mg/L)								
			N1	N3	N5	N8	MO36	MO56	PF2	PM32	PM52
Esters	Ethyl acetoacetate	et1	34,232.52 ± 0.03606^e^	21,022.43 ± 0.04000^c^	47,024.66 ± 0.04359^i^	39,197.32 ± 0.04000^g^	33,576.2 ± 0.04359^d^	21,010.92 ± 0.05568^a^	21,015.8 ± 0.1000^b^	47,019.86 ± 0.02646^h^	39,185.26 ± 0.04359^f^
	Isoamyl acetate	et2	78.11 ± 0.03612^e^	72.26 ± 0.05000^a^	82.83 ± 0.03606^f^	138.35 ± 0.03606^i^	76.6 ± 0.20000^d^	72.47±0.02646^b^	73.36 ± 0.02000^c^	84.94 ± 0.03606^g^	125.24 ± 0.04583^h^
	Butyl acetate	et3	24.74 ± 0.03626^a^	19.28 ± 0.02644^c^	15.92 ± 0.02646^e^	10.98 ± 0.02010^g^	24.9 ± 0.04509^b^	19.26 ± 0.02000^c^	19.02 ± 0.05568^d^	15.05 ± 0.03464^f^	9.8 ± 0.03689^h^
	Ethyl‐2‐hydroxypropanoate	et4	102.26 ± 0.03000^c^	606.62 ± 0.03001^i^	318.41 ± 0.02000^f^	87.47 ± 0.02000^b^	103.2 ± 0.20000^d^	605.36 ± 0.02154^g^	605.79 ± 0.02646^h^	316.6 ± 0.2000^e^	85.51 ± 0.04359^a^
	Ethyl butyrate	et5	55.95 ± 0.03551^d^	101.6 ± 0.20000^h^	73.74 ± 0.02464^f^	41.55 ± 0.04000^b^	54.2 ± 0.10000^c^	100.78 ± 0.04359^g^	102.56 ± 0.02103^i^	69.19 ± 0.02109^e^	41.07 ± 0.06028^a^
	Ethyl valerate	et6	3.53 ± 0.01732^b^	36.74 ± 0.02000^h^	18.59 ± 0.03000^f^	10.69 ± 0.02646^c^	3.41 ± 0.01011^a^	36.52 ± 0.08185^g^	38.6 ± 0.04359^i^	16.3 ± 0.03000^e^	10.8 ± 0.03010^d^
	Ethyl hexanoate	et7	41.34 ± 0.02646^a^	62.79 ± 0.03464^f^	60.68 ± 0.02664^c^	63.17 ± 0.02000^g^	41.75 ± 0.02646^b^	63.56 ± 0.04000^h^	63.86 ± 0.02646^i^	62.17 ± 0.04583^d^	62.69 ± 0.05000^e^
	Ethyl heptanoate	et8	0.77 ± 0.02764^b^	1.09 ± 0.03000^d^	1.28 ± 0.02000^h^	1.98 ± 0.04000^i^	0.69 ± 0.0500^a^	1.09 ± 0.0100^e^	1.14 ± 0.01732^f^	1.19 ± 0.01000^g^	1.07 ± 0.02646^c^
	Ethyl lactate	et9	273,354.73 ± 0.04359^d^	219,489.21 ± 0.02646^c^	339,131.71 ± 0.17321^f^	382,868.37 ± 0.04359^h^	276,114.6 ± 0.17321^e^	219,385.54 ± 0.30000^a^	219,386.18 ± 0.3650^b^	339,134.55 ± 0.04000^g^	392,575.98 ± 0.01000^i^
	Ethyl octanoate	et10	6.86 ± 0.02646^a^	11.17 ± 0.02641^e^	9.71±0.02649^b^	15.46 ± 0.03606^h^	6.91 ± 0.03606^a^	11.08 ± 0.02646^d^	11.98 ± 0.03000^f^	10.39 ± 0.05000^c^	12.08 ± 0.03606^g^
	Diethyl succinate	et11	11,686.06 ± 0.03464^b^	9,050.72 ± 0.03606^i^	9,410.81 ± 0.04000^f^	12,193.07±0.02646^a^	11,179.6 ± 0.45826^c^	9,061.07 ± 0.02001^h^	9,061.67 ± 0.20000^g^	9,411.73 ± 0.03604^e^	9,859.31 ± 0.04359^d^
	Ethyl phenylacetate	et12	18.8 ± 0.04359^d^	15.83 ± 0.02000^g^	33.95 ± 0.03607^b^	8.16 ± 0.04359^i^	18.89 ± 0.04000^c^	16.65 ± 0.02646^e^	16.05 ± 0.03754^f^	34.6 ± 0.26458^a^	8.87 ± 0.05292^h^
	Phenethyl acetate	et13	16.72 ± 0.03606^d^	10.99 ± 0.01000^a^	22.16 ± 0.02000^f^	26.43 ± 0.04359^h^	16.82 ± 0.01000^e^	11.54 ± 0.02646^b^	11.98 ± 0.03000^c^	25.87 ± 0.05292^g^	28.26 ± 0.04359^i^
	γ‐nonanoic lactone	et14	30.73 ± 0.02646^a^	43.45 ± 0.03644^e^	61.24 ± 0.02443^f^	64.69 ± 0.06083^i^	31.5 ± 0.10000^b^	43.41 ± 0.04000^d^	43.24 ± 0.02646^c^	62.17 ± 0.02000^g^	64.25 ± 0.05000^h^
	Ethyl palmitate	et15	55.16 ± 0.03465^e^	263.11 ± 0.04000^g^	27.21 ± 0.02000^a^	33.23 ± 0.03235^c^	55.6 ± 0.20000^f^	265.14 ± 0.03606^h^	265.45 ± 0.04583^i^	28.61 ± 0.05022^b^	34.92 ± 0.05196^d^
Alcohols	2‐methyl‐1‐propanol	al1	71,538.18 ± 0.03606^c^	94,908.73 ± 0.04583^g^	99,887.34 ± 0.04359^i^	59,573.77 ± 0.09000^a^	72,196.58 ± 0.06928^d^	94,807.75 ± 0.07000^e^	94,903.78 ± 0.05000^f^	95,911.95 ± 0.06000^h^	59,601.15 ± 0.03000^b^
	2‐Methyl‐1‐butanol	al2	1572.05 ± 0.04000^a^	3,904.39 ± 0.06083^c^	1938.82 ± 0.57239^b^	4,165.19 ± 0.03606^d^	1586.5 ± 0.09000^a^	3,918.79 ± 0.07211^c^	3,918.62 ± 0.04583^c^	1940.01 ± 0.01583^b^	4,164.04 ± 0.01154^d^
	3‐methyl‐1‐butanol	al3	256,856.8 ± 0.52915^c^	258,145.54 ± 0.04359^e^	304,930.98 ± 0.12530^h^	250,433.25 ± 0.06557^b^	259,333.2 ± 0.36056^g^	257,948.16 ± 0.06245^d^	258,146.66 ± 0.05292^f^	305,110.06 ± 0.04359^i^	229,439.04 ± 0.0781^a^
	2‐Heptanol	al4	1.39 ± 0.05000^a^	1.93 ± 0.07550^g^	5.17 ± 0.03646^h^	1.69 ± 0.04359^d^	1.39 ± 0.03606^b^	1.84 ± 0.07000^e^	1.9 ± 0.04359^f^	6.05 ± 0.04000^i^	1.68 ± 0.07000^c^
	Hexanol	al5	100.57 ± 0.02000^c^	206.86 ± 0.01000^f^	293.53 ± 0.06245^h^	82.48 ± 0.08544^b^	100.56 ± 0.06083^c^	205.5 ± 0.13229^d^	206.05 ± 0.05568^e^	292.680 ± 0.07000^g^	80.87 ± 0.07937^a^
	Furfuryl alcohol	al6	641.79 ± 0.04359^c^	553.04±0.052920^e^	804.09 ± 0.01000^b^	200.08 ± 0.02646^h^	638.97 ± 0.02646^d^	552.05 ± 0.05292^g^	552.62 ± 0.02646^f^	814.87 ± 0.04359^a^	199.41 ± 0.05292^i^
	2‐Phenylethanol	al7	320,771.43 ± 0.03000^e^	280,194.37 ± 0.05568^c^	315,379.15 ± 0.04583^d^	364,104.95 ± 0.06000^h^	323,933.58 ± 0.07550^f^	280,170.59 ± 0.03606^a^	280,189.11 ± 0.04359^b^	335,483.41 ± 0.04355^g^	364,816.82 ± 0.03606^i^
	2,3‐Butanediol	al8	1724.05 ± 0.03658^a^	2,613.90 ± 0.05292^f^	1987.55 ± 0.05568^c^	3,781.14 ± 0.04359^i^	1732.01 ± 0.08000^b^	2,611.92 ± 0.02646^e^	2,615.18 ± 0.16093^g^	1989.41 ± 0.05568^d^	3,777.05 ± 0.08000^h^
Aldehyde	Furfural	ad1	4,582.03 ± 0.02000^a^	7,916.2974 ± 0.00140^d^	12,397.82 ± 0.05292^f^	15,337.92 ± 0.03606^i^	4,628.21 ± 0.05568^b^	7,915.73 ± 0.05292^c^	7,918.51 ± 0.05298^e^	12,401.53 ± 0.04359^g^	15,327.2 ± 0.04359^h^
	Benzaldehyde	ad2	922.72 ± 0.04000^d^	458.18 ± 0.06245^f^	440.27 ± 0.03464^i^	2,150.63 ± 0.03606^a^	931.6 ± 0.05196^c^	455.52 ± 0.04000^g^	458.59 ± 0.04000^e^	445.62 ± 0.04000^h^	2,130.79±0.02646^b^
	5‐Methyl furfural	ad3	9.98 ± 0.05292^b^	13.93 ± 0.03606^d^	39.65 ± 0.03606^f^	112.2 ± 0.11547^i^	9.94 ± 0.04359^a^	12.13 ± 0.05292^c^	13.94 ± 0.07000^e^	41.78 ± 0.03606^g^	111.54 ± 0.06557^h^
	Phenylacetaldehyde	ad4	159.67 ± 0.04000^f^	139.74 ± 0.05196^h^	221.47 ± 0.02000^b^	193.79 ± 0.05292^c^	160.93 ± 0.02646^e^	138.62 ± 0.03606^i^	140.18 ± 0.06557^g^	222.06 ± 0.06083^a^	191.49 ± 0.03606^d^
Acids	Nonanoic acid	ac1	424.38 ± 0.04359^a^	366.82 ± 0.03646^c^	346.71 ± 0.05292^d^	285.7 ± 0.04359^i^	420.5 ± 0.36056^b^	342.64 ± 0.04359^e^	341.32 ± 0.02646^f^	298.97 ± 0.02000^g^	286.12 ± 0.02646^h^
	Isovaleric acid	ac2	199.99 ± 0.05000^f^	143.60 ± 0.05568^i^	256.07 ± 0.05196^d^	278.94 ± 0.02646^a^	201.87 ± 0.02596^e^	145.33 ± 0.02646^h^	146.03 ± 0.07000^g^	256.5 ± 0.04359^c^	266.81 ± 0.05292^b^
	Hexanoic acid	ac3	105.92 ± 0.02646^a^	239.32 ± 0.03606^f^	218.41 ± 0.05292^b^	236.44 ± 0.07810^e^	105.8 ± 0.05292^a^	239.26 ± 0.05568^f^	240.18 ± 0.06557^g^	218.77 ± 0.04359^c^	234.5 ± 0.26458^d^
	Octanoic acid	ac4	28.81 ± 0.01732^g^	29.54 ± 0.07550^e^	60.71 ± 0.05196^b^	53.69 ± 0.03000^c^	28.61 ± 0.05000^h^	27.88 ± 0.05568^i^	28.9 ± 0.04000^f^	62.17 ± 0.07550^a^	53.66 ± 0.06083^d^
	Ethyl heptanoate	ac5	194.59 ± 0.04000^b^	106.83 ± 0.05292^g^	175.53 ± 0.03606^c^	155.06 ± 0.04359^e^	195.25 ± 0.04357^a^	104.54 ± 0.04583^i^	106.7 ± 0.6245^h^	177.11 ± 0.05292^d^	144.14 ± 0.04359^f^
Phenols	Phenol	pl1	58.60 ± 0.20000^b^	43.81 ± 0.07000^c^	36.62 ± 0.04583^h^	43.33 ± 0.03464^e^	58.9 ± 0.02646^a^	42.91 ± 0.07211^f^	43.51 ± 0.04000^d^	34.65 ± 0.03606^i^	41.54 ± 0.04583^g^
	Total		979,601.23	900,794.1174	1,135,712.79	1,135,951.17	987,569.33	900,345.55	900,688.48	1,152,000.82	1,122,972.96

Note

Values are significantly different (*p* <.05) labeled by different superscript letters within a column.

**FIGURE 2:**
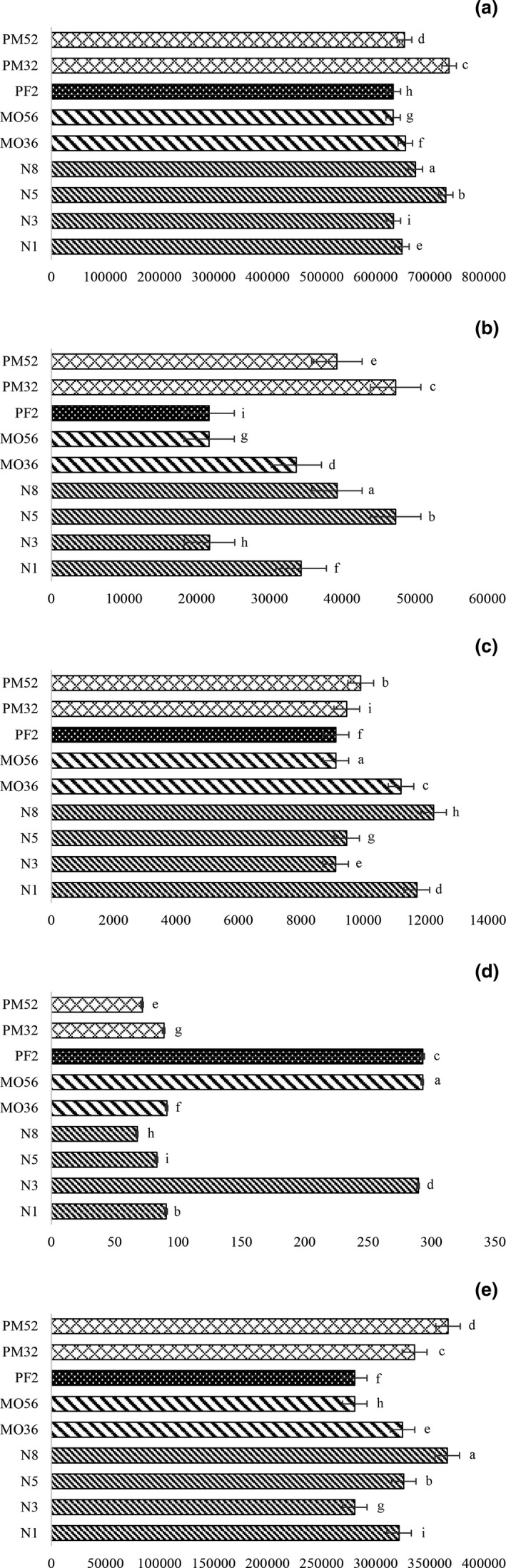
Total contents of main flavoring substances in Huangjiu under different treatment conditions. (a) Major alcohols (2‐methyl‐1‐propanol, 3‐methyl‐1‐butanol, 2‐Phenylethanol). (b) SCFAA (ethyl acetoacetate, ethyl butyrate, ethyl‐2‐hydroxypropanoate). (C) MCFAEE (ethyl hexanoate, ethyl heptanoate, ethyl octanoate, diethyl succinate). (d) LCFAEE (ethyl phenylacetate, phenethyl acetate, ethyl palmitate). (e) Aromatic compounds (2‐phenylethanol, phenethyl acetate, ethyl phenylacetate, γ‐nonanoic lactone, benzaldehyde, phenylacetaldehyde). In each figure, mean values with different letters are differed significantly by Duncan's test (*p* <.05)

Huangjiu contained a large number of volatile flavor compounds, the most abundant were alcohols, esters, and aldehydes, which constitute the structural components of the aroma of Huangjiu (Rang et al., 2016; Ren et al., 2019). Oxidation, reduction, esterification, and hydrolysis reactions occurred in wine, influencing the contents and numbers of alcohols, aldehydes and esters (Tian et al., 2016). After aging, most alcohols in Huangjiu were on the rise, and the content of N5 (735,226.63 mg/L) fluctuated in 5 years (Table 3). 2‐methyl‐1‐propanol, 3‐methyl‐1‐butanol, and 2‐phenylethanol, which accounted for more than 95% of the total content of alcohols, were the key alcohols identified in Huangjiu (Xu et al., 2015). The total content of those three alcohols in different Huangjiu is shown in Figure 2. The three alcohols fluctuated during the natural aging process, and the other two alcohols, except 2‐Phenylethanol, showed a decreasing trend. The content of the three alcohols was increased after the treatment of micro‐oxygen and electric field, among which the trend of 2‐phenylethanol increased obviously. 2‐Phenylethanol, as the most abundant compound in alcohols, was greatly affected (Osada et al., 2015). It is mainly produced through the Ehrlich pathway, which transforms L‐phenylalanine into 2‐Phenylethanol by aminotransferase, decarboxylase, and dehydrogenase (Ivanov et al., 2013). Compared with Huangjiu treated with electric field only, the content of phenylalanine in PM52 and PM32 was relatively low (Figure 1c), which could indirectly explain the results. Huangjiu at the electric field intensity of 2 kv/cm, the micro‐oxygen is over 0.5 mg L/day, could activate enzymes and contribute to the synthesis of 2‐phenylethanol (Figure 2a).

The results showed that the total ester content of Huangjiu treated with micro‐oxygen or electric field was equivalent to that of natural aging. The total ester loss of natural aging was faster in the initial storage, with a reduction of 21.55% (Table 3). Most of the esters were fatty acid ethyl esters (FAEE), including short‐chain (SC, C2–C5), medium‐chain (MC, C6–C12), and long‐chain (LC, C13–C18) FAEE (Jung et al., 2014; Mo et al., 2012). Compared with natural aging, the content of SCFAEE decreased after micro‐oxygen treatment, while that of LCFAEE increased. The MCFAEE content of PM52 and PM32 were similar to that of 3‐year natural aging and 5‐year natural aging. Natural aging was beneficial to the increase of ester content. During the aging process, the content of SCFAEE and MCFAEE increased by 12.55% and 4.39%, respectively, while the content of LCFAEE decreased by 25.21% (Table 3). In the Huangjiu treated with micro‐oxygen and electric field, with the increment of oxygen content from 0.35 mg L/day to 0.5 mg L/day, the content of SCFAEE in the 2 kv/cm electric field treatment decreased by 17.07%, while in the Huangjiu treated with micro‐oxygen treatment, the content of SCFAEE decreased by 35.63% (Figure 2b, Table 3). The MCFAEE content of PM53 and PM32 is higher than that of N5, while the MCFAEE content of PF2 is higher than that of N3 (Figure 2c, Table 3). LCFAEE content of N8 decreased by 25.51% compared with N1, and the LCFAEE in PF2 and MO56 was close to that in N3 (Figure 2d, Table 3). It is worth noting that the high content of LCFAEE has a great influence on the results.

The aromatic compounds in wine aroma included 2‐phenylethanol, ethyl phenylacetate, phenylethyl acetate, benzaldehyde, and benzeneacetaldehyde in Huangjiu (Qiao et al., 2016). The content of aromatic compounds in PM52 (367,240.48 mg/L) was the highest, followed by PM32, PF2, and MO36 (Figure 2e). The contents of aromatic compounds among accelerated aging‐treated Huangjiu were significantly different, especially PM52. 2‐Phenylethanol, which has a floral scent, greatly affects the total contents of aromatic compounds. PM52 contained the highest content of 2‐phenylethanol (364,816.82 mg/L), which was about 12.07% higher than N1. At 0.5 mg L/day 2 kv/cm, and micro‐oxygen and electric field were most beneficial to the growth of the total content of aromatic compounds.

The results showed that FAEE in Huangjiu treated with micro‐oxygen and electric field could reach the level of natural aged Huangjiu, especially in MCFAEE and LCFAEE. Accelerated aging treatments achieve natural aging levels in the production of 2‐methyl‐1‐propanol, 3‐methyl‐1‐butanol, 2‐phenylethanol, and aromatic compounds.

### 3.4 Effects on flavor characteristic of Huangjiu

**FIGURE 3:**
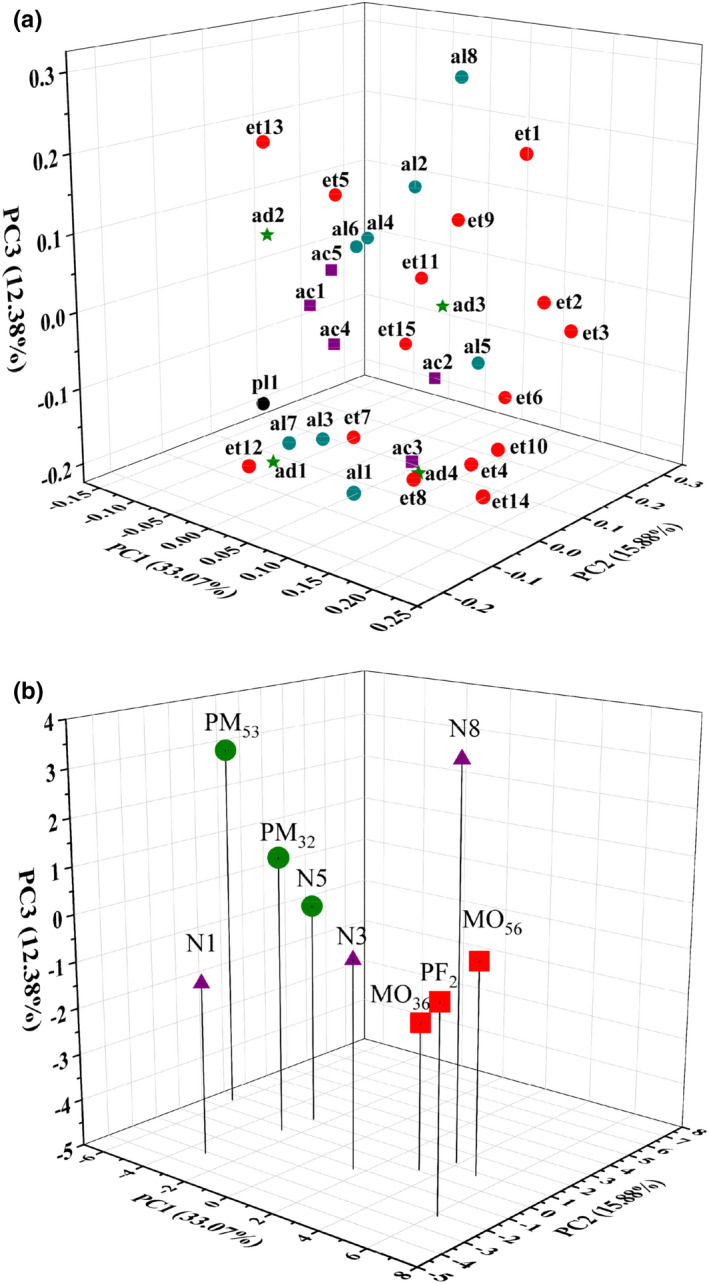
Loadings plot for the 33 volatile flavor compounds (a) and different treatments of Huangjiu scores plot (b)

The main component analysis (PCA) was used to analyze the flavor characteristics of Huangjiu, and 33 volatile flavor components were used as analysis variables. PCA could reduce the dimensionality within the data set and detect similarities and/or differences between wine samples. As shown in Figure 3, PCA revealed the relationship between flavor compounds and different Huangjiu.

The results shown in Figure 3a revealed loading plots, whereby the first, second, and third principal component (PC), respectively, explained 33.07%, 15.88%, and 12.38% of the total variance. Thirty‐three compounds positively loaded on PC1, which was highly influenced by isoamyl acetate, diethyl succinate, phenylethyl acetate, benzaldehyde, phenylacetaldehyde, and hexanoic acid. The negative axis of PC1 was closely related to 3‐methyl‐1‐butanol, 2‐phenylethanol, ethyl hexanoate, ethyl benzoate, and furaldehyde. The contents of MCFAEE, LCFAEE, and aromatic compounds greatly affect the flavor characteristics of analyzed Huangjiu.

The distribution of scores in the 9 wines (Figure 3b) revealed three separate groups. PM32 and PM52 were close to the flavor characteristics of N5, which indicated that micro‐oxygen and electric field treatment could shorten the formation time of flavoring substances in Huangjiu. Group 2, formed by MO36, MO56, and PF2, showed high levels on the negative side of PC1 and positive side of PC3. Flavor characteristics of Huangjiu in Group 2 were mainly affected by the presence of 3‐methyl‐1‐butanol, 2‐phenylethanol, furfural, ethyl hexanoate, and ethyl phenylacetate. The high content of 3‐methyl‐1‐butanol and 2‐phenylethanol has a great influence on the flavor characteristics of Huangjiu after accelerated aging treatment. Group 3 was made up of N1, N3, and N8, which were found with low values in N1. 2‐Methyl‐1‐propanol, hexanoic acid, octanoic acid, ethyl heptanoate, ethyl‐2‐hydroxypropanoate, ethyl octanoate, γ‐nonanoic lactone, and phenylacetaldehyde played predominant roles in the flavor characteristics of Huangjiu in Group 3.

### 3.5 Sensory evaluation

**FIGURE 4:**
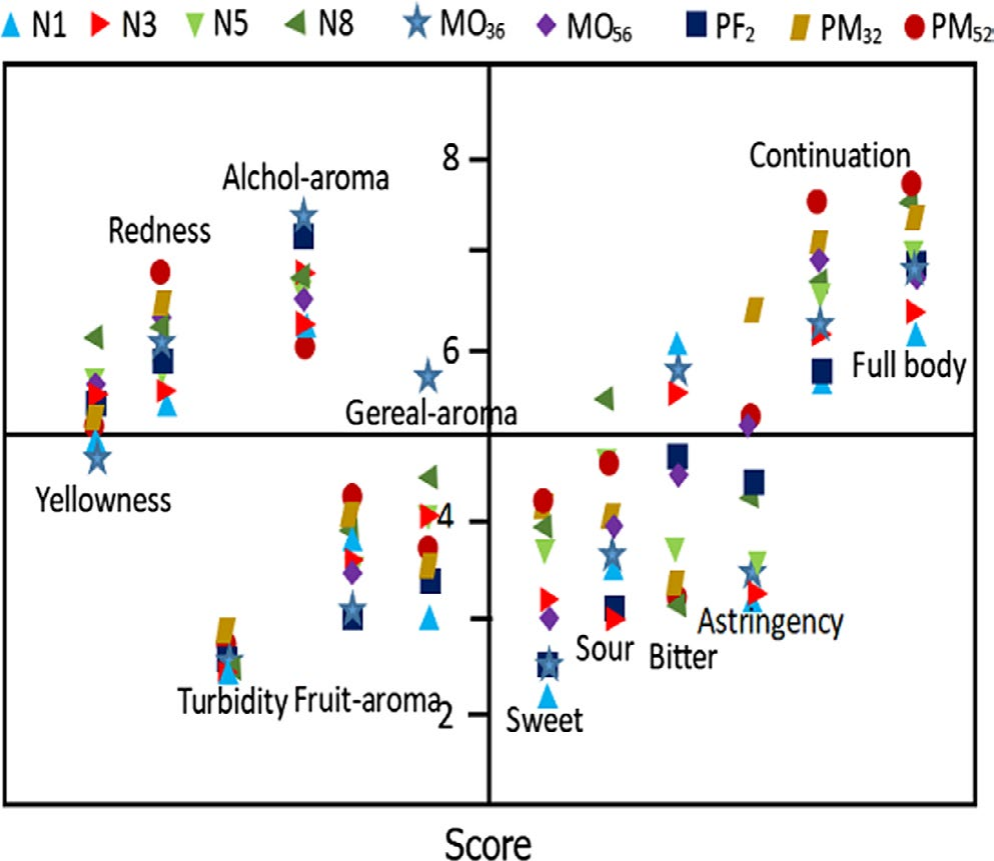
Scores plot of sensorial attributes for Huangjiu processed by different treatments

To profile the sensory profiles of Huangjiu, descriptive sensory analysis was conducted. The average strength levels of natural aging, micro‐oxygen treatment, and electric field treatment are shown in Figure 4. Natural aging exhibited a high level of yellowness, while PM52 showed high intensity of redness, which was in agreement with the color analysis results (Figure 1). Micro‐oxygen and electric field‐treated wines exhibited higher levels in redness than natural aging, suggesting appropriate electric field treatment reduced the loss of red pigment. All 9 wines exhibited similar clarity in turbidity, which were all scored lower than 3. Turbidity index of the nine samples ranged from 0.7 to 0.9 NTU, which exhibited a good clarification. Different processing had little effect of Huangjiu. MO36 had the highest alcohol‐aroma index and PM52 had the lowest alcohol‐aroma index, which was consistent with the determination of alcohol content (Table 3). Esters with pleasant aroma were responsible for the perception of fruit aroma. Except for PM52 which scored higher than 4 in fruit aroma, other treatments rice wine were assessed with low intensity in fruit aroma. In terms of cereal aroma, MO36 score was the highest among the treatment Huangjiu, followed by PM52 and PM32, and the score of Huangjiu treated with micro‐oxygen was significantly higher than that of naturally aged Huangjiu. Except that the scores of PM32 and PM52 were higher than 4, all other indexes of sweet taste were improved. The unpleasant attributes in N1 (relative to the industry), where astringency intensity increased slightly with aging time and bitterness intensity decreased. PM52 was intense in fruit aroma, mouthfeel of continuation, and full body (pleasant attributes relative to the industry) and was the most highly assessed Huangjiu among the nine wines. Among naturally aged Huangjiu, N8 scored highest for pleasant properties and lowest for unpleasant ones, meaning that the closer the effect was to N8, the better the flavor. However, in contrast to traditional aged wines, micro‐oxygen with electric field‐treated wines presented a more mellow taste which was preferred by the panelists.

### 3.6 Relationship between sensory attributes and flavor compounds

**FIGURE 5:**
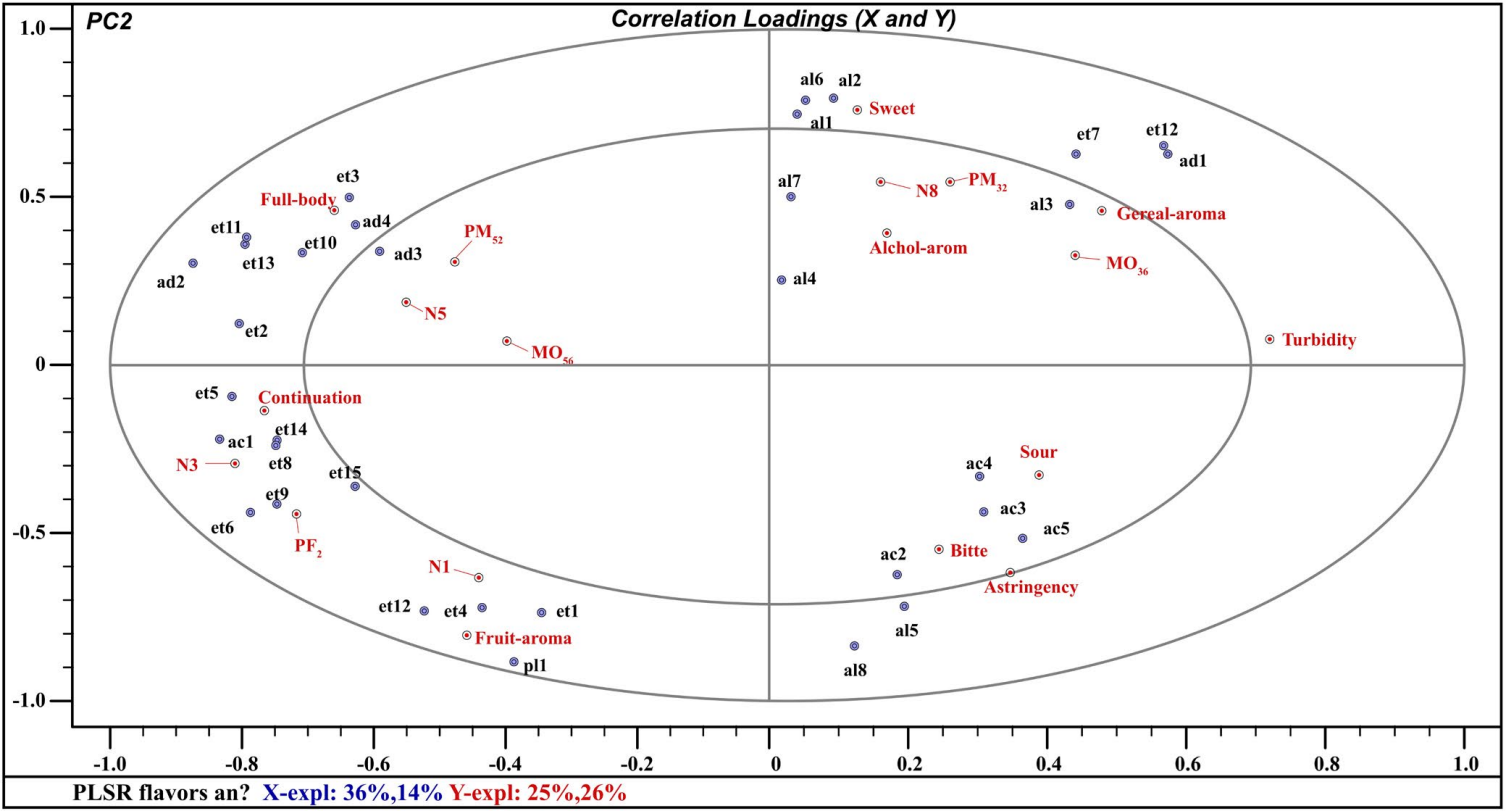
Load diagram of sensory indexes and 33 volatile components of Huangjiu under natural aging and accelerated aging treatment. (The codes were defined in Table 3, respectively)

Multivariate PLSR was performed to study the relationship between sensory attributes and volatile compounds. All volatile flavor compounds and nine sensorial attributes (fruit aroma, cereal aroma, alcohol aroma, sweet, astringency, sour, bitter, full body, and continuation) were designed as dependent variables. PLSR modeling between the matrices of volatile compounds and sensory attributes provided a two‐factor model explaining 50% of the variance in X (volatile compounds) and 51% of that in Y (sensory attributes). The small ellipse indicated 50% of the explained variance, and the big ellipse is the unit‐circle indicating 100% of the explained variance (Xiao et al., 2014). Aroma compounds between the two ellipses could be considered as correlated with sensory attributes, and those inside the inner ellipse were poorly connected with sensory attributes. Eleven Y variables (N1, N3, N5, N8, PF2, MO36, MO56, PM32, PM52, full body, continuation, fruit aroma, astringency, cereal aroma, and sweet) and thirty‐three X variables were located between the inner and outer ellipses (Figure 5), indicating they were well explained by the PLSR model.

As shown in Figure 5, MO36 correlated well with cereal aroma, which was in agreement with the sensory evaluation results (Figure 4), where the micro‐oxygen treated Huangjiu had high scores in cereal aroma. The cereal aroma was closely related to ethyl hexanoate, ethyl phenylacetate, and furfural. The astringency characteristics of the three Huangjiu in the right quadrant indicate that their astringency will become prominent after aging. N1 and N3, located in the lower‐left quadrant, were associated with fruit aroma and continuation. This result is in good agreement with PCA and sensory evaluation. The two kinds of Huangjiu have strong fruit aroma and continuity. The attribute of fruit aroma showed high correlation with ethyl acetate, ethyl‐2‐hydroxypropanoate, and ethyl phenylacetate. Although MO56 and full body were all in the upper‐left quadrant, poor association was observed between full body and the two wines. Full body was pleasant for customers, and it was highly correlated with ethyl octanoate, diethyl succinate, phenylethyl acetate, and benzaldehyde. PM32 and N8 in the upper‐right quadrant correlated with attributes of sweet and alcohol aroma. Sweet taste showed little correlation with any flavor compounds and alcohol aroma was associated with 3‐methyl‐1‐butanol, 2‐heptanol, and 2‐phenylethanol. The above results revealed that aromatic compounds, MCFAEE, and LCFAEE made great contributions to the pleasant attributes (full body, continuation) of Huangjiu. Therefore, sensory characteristics of Huangjiu could be enhanced by improving the contents of aromatic compounds, MCFAEE, and LCFAEE. After aging treatment, MCFAEE and LCFAEE in PM52 can reach the natural aged 5 years, which is 11.01% higher than the natural aged 5 years.

## 4 CONCLUSIONS

The effects of traditional natural aging, micro‐oxygen and electric field‐ treatments on oenological properties, free amino acid, flavor characteristics, and sensory profiles were investigated. After aging, the brewing characteristics of Huangjiu changed significantly, but the amino content could reach the level of natural aged Huangjiu after being treated with micro‐oxygen and electric field. The Huangjiu treated with 0.35 mg L/day or 0.5 mg L/day combined with electric field 2 kv/cm showed similar effects, and the flavor characteristics of naturally aged Huangjiu were compared with those of naturally aged Huangjiu, and it was found that the flavor characteristics of naturally aged Huangjiu were closely related to CFAEE. The sensory profiles of Huangjiu could be enhanced by improving the contents of aromatic compounds, MCFAEE, and LCFAEE. In this experiment, the aging treatment of 0.5 mg L/day and 2 kv/cm could maximize the sensory profiles. Because natural aging has a significant effect on free amino acids, flavor characteristics, and sensory characteristics, our results suggest that micro‐oxygen, electric field treatments can be used instead. Among them, the flavor characteristics of Huangjiu treated with micro‐oxygen are similar to those of naturally aged Huangjiu, while the combination of electric field treatment can improve the flavor intensity of fermented Huangjiu to a greater extent.

## AUTHOR CONTRIBUTIONS

Chi Shen: Formal analysis (equal). Hongyi Zhu: Conceptualization (equal); Data curation (equal); Methodology (equal); Project administration (equal); Writing‐original draft (equal). Wenxia Zhu: Conceptualization (equal); Methodology (equal); Project administration (equal); Writing‐original draft (equal). Yimeng Zhu: Software (equal). Qi Peng: Conceptualization (equal); Funding acquisition (equal); Project administration (equal); Supervision (equal). Nabil I. Elsheery: Investigation (equal). Jianwei Fu: Validation (equal). Guangfa Xie: Formal analysis (equal). Huajun Zheng: Visualization (equal). Jiongping Han: Writing‐review & editing (equal). Baowei Hu: Validation (equal). Jianqiu Sun: Validation (equal). Peng Wu: Resources (equal). Yuyan Fan: Resources (equal). Dula Bealu Girma: Visualization (equal).

### 1 DATA AVAILABILITY STATEMENT

The data of this study are openly available.
